# Histone modification and chromatin remodeling in plant response to pathogens

**DOI:** 10.3389/fpls.2022.986940

**Published:** 2022-10-03

**Authors:** Huijia Kang, Tianyi Fan, Jiabing Wu, Yan Zhu, Wen-Hui Shen

**Affiliations:** ^1^State Key Laboratory of Genetic Engineering, Collaborative Innovation Center for Genetics and Development, Department of Biochemistry, Institute of Plant Biology, School of Life Sciences, Fudan University, Shanghai, China; ^2^Ministry of Education Key Laboratory for Biodiversity Science and Ecological Engineering, Institute of Biodiversity Science, School of Life Sciences, Fudan University, Shanghai, China; ^3^Institut de Biologie Moléculaire des Plantes (IBMP), CNRS, Université de Strasbourg, Strasbourg, France

**Keywords:** epigenetics, histone modification, chromatin remodeling, plant defense, pathogen infection

## Abstract

As sessile organisms, plants are constantly exposed to changing environments frequently under diverse stresses. Invasion by pathogens, including virus, bacterial and fungal infections, can severely impede plant growth and development, causing important yield loss and thus challenging food/feed security worldwide. During evolution, plants have adapted complex systems, including coordinated global gene expression networks, to defend against pathogen attacks. In recent years, growing evidences indicate that pathogen infections can trigger local and global epigenetic changes that reprogram the transcription of plant defense genes, which in turn helps plants to fight against pathogens. Here, we summarize up plant defense pathways and epigenetic mechanisms and we review in depth current knowledge’s about histone modifications and chromatin-remodeling factors found in the epigenetic regulation of plant response to biotic stresses. It is anticipated that epigenetic mechanisms may be explorable in the design of tools to generate stress-resistant plant varieties.

## Plant pathways against pathogens

Naturally growing plants are inevitably exposed to attacks by a variety of harmful pathogens. Invasions by viruses and bacteria rely on the natural openings or damaged tissues of plants, while other pathogens such as oomycetes and fungi can directly penetrate the surface of host plants. Resistance usually manifests itself as a multi-layered defense, which includes the barrier formed by cell wall as well as by the waxy and cuticle layers on the surface of plant organs, which naturally prevents the colonization of pathogens. Importantly plants also have evolved divers defense-responsive mechanisms that are induced upon pathogen attack, such as physical reinforcements of cell walls by generating callose and lignin, and phytoanticipins and antimicrobial metabolites/proteins productions (Gill et al., 2015). Meanwhile the activation of defense responses may have a detrimental effect on plant growth and thus needs to be tightly regulated to strict necessities.

Plants have developed complex sensory mechanisms to identify pathogen invasions (Jones and Dangl, 2006). As a primary step of defense response, plants widely use receptor kinases and receptor-like proteins located on the plasma membrane, namely pattern-recognition receptors (PRRs), which sensitively recognize pathogen-associated molecular patterns (PAMPs) / microbe-associated molecular patterns (MAMPs; e.g., flg22) or damage-associated molecular patterns (DAMPs; e.g., endogenous peptide 1; Boutrot and Zipfel, 2017). Such recognition triggers a range of downstream defense mechanisms that lead to the activation of pattern-triggered immunity (PTI; Bigeard et al., 2015). PTI involves physiological phenomena with distinctive characteristics, such as stomatal closure and callose deposition to limit pathogen penetration, production of reactive oxygen species (ROS) and nitric oxide (NO), limitation of nutrient transfer from the cytoplasmic matrix to apoplast, and biosynthesis of antimicrobial metabolites and defense phytohormones to fight against pathogen invasion and propagation.

Meanwhile, some well-adapted pathogens have evolved to produce specific effectors and deliver them into host plant cells (Nguyen et al., 2021). Many of these pathogenic bacteria complete this physiological process through the Type III secretion system. Such effectors prevent the host from recognizing PAMPs/MAMPs and deregulate immune signaling and expression, leading to effector-triggered susceptibility (ETS; Feng and Zhou, 2012; Schreiber et al., 2021; Hannan Parker et al., 2022).

During evolution, plants have evolved specific *R* (resistance) genes encoding proteins that can detect the activity of immune-inhibitory effectors, and pathogens co-evolve new effectors to evade or counteract the plant detection. Plant *R* genes typically encode intracellular receptors with nucleotide-binding leucine-rich repeat (NB-LRR) domains, organized with central NB and C-terminal LRRs. These NB-LRR-containing R proteins (NLRs) can be further classified into Toll/Interleukin1 receptor-like (TIR) or coiled-coil (CC) types according to their N-terminal sequences. NLRs can interact directly or indirectly with pathogen effectors to induce defense responses (Spoel and Dong, 2012; Nguyen et al., 2021). One of the *R* gene-mediated resistance responses is known as oxidative burst that rapidly produce ROS, which may have a direct antimicrobial effect and/or acts as a signal to activate other defense responses. The other well-known defense response is called hypersensitive response (HR) that is associated with local cell death to limit the pathogen propagation. *R* gene-mediated resistance is thought to be related to the activation of phytohormone salicylic acid (SA)-dependent signaling pathways, in which most R proteins participate in ROS production and HR development, leading to effector-triggered immunity (ETI; Nguyen et al., 2021).

Although the extent and duration of the immune response triggered by different pathogen effectors vary greatly during PTI and ETI, plasma membrane-localized immune receptors activate similar downstream molecular events, such as activation of mitogen-activated protein kinase (MAPK), Ca^2+^ burst (Koster et al., 2022), oxidative burst, ion influx, and increased biosynthesis of plant defense hormones, suggesting that the defensive signals initiated on the plasma membrane converge in the downstream pathway (Iakovidis et al., 2022). PTI and ETI pathway-induced signaling share multiple proteins, such as transcription factors CALMODULIN-BINDING PROTEIN 60 g (CBP60g) and SYSTEMIC ACQUIRED RESISTANCE DEFICIENT 1 (SARD1), in which the two proteins bind directly to the promoter of the SA-synthase genes and activate their expression, which in turn leads to the activation of immune-related genes, such as *PATHOGENESIS-RELATED GENE 1* (*PR1*) and *PR2* (Peng et al., 2018).

While effective defense against biotrophic pathogens that feed on living host tissues primarily relies on the activation by SA-dependent pathway and *R* gene-mediated resistance, necrotrophic pathogens that kill host tissue and feed on the remains need to be counteracted by a different set of defense responses activated by jasmonic acid (JA) and ethylene (ET) signaling pathways. As a result, the SA, JA, and ET signal pathways have an extensive interaction. SA and JA have mutual inhibitory effects on the expression of many defense genes. It is widely believed that this antagonism between SA and JA enables plants to be more effective against different types of pathogens, with SA-mediated signaling pathways involved in the defense against biotrophic pathogens, while JA-mediated signaling participates in defense against necrotrophic pathogens (Ghorbel et al., 2021; Peng et al., 2021).

Treatments on genetically susceptible plants by inducing molecules (such as JA) can improve plant innate immunity levels and enhance plant defenses against upcoming pathogens and pests (Luna et al., 2016). This so-called induced resistance (IR) is often considered as an adaptive component of plant immune system as well as a form of phenotypic plasticity (Mauch-Mani et al., 2017; De Kesel et al., 2021). IR is the sum of direct and primed defense responses. Priming refers to an enhanced capacity where the postchallenged/posttreated state mounts a faster and/or stronger defense response to the upcoming attack. A typical example of IR is systemic acquired resistance (SAR) where upon local pathogen attack plants develop strong resistance in distal tissues to against subsequent attacks (Gao et al., 2021). SAR is activated by signal substances produced at the site of infection. Many endogenous metabolites have been shown to be potential elicitors for SAR, including methyl salicylate (MeSA) and free radicals (such as NO and ROS; Gao et al., 2021). These small signaling molecules can be transported to distal tissues through phloem, resulting in hormone SA accumulation and PR proteins secretion to induce SAR. Thus, over the course of weeks to months, the rest of host plant can gain a strong defense response and broad-spectrum disease resistance through SAR against secondary infections (Fu and Dong, 2013). Arabidopsis (*Arabidopsis thaliana*) NON-EXPRESSER OF *PR*-GENES 1 (NPR1) protein was identified as the main regulator of SAR (Luna et al., 2012). In general, proper transcriptional programming/reprogramming is widely believed to be critical for priming and plant defense against multiple pathogens (Tsuda and Somssich, 2015; Birkenbihl et al., 2017; Mauch-Mani et al., 2017).

## Epigenetic regulation in plant response to pathogens

Epigenetics refers to heritable changes in gene expression that do not involve alterations of DNA sequences. Epigenetic regulation relies on changing chromatin structures. The basic unit of chromatin is nucleosome, which is composed by approximately 146 bp of DNA wrapped around a histone octomer, comprising two molecules of each of histones H2A, H2B, H3 and H4 (Luger et al., 1997). Nucleosomes are joined by variable-length internucleosomal DNA and the linker histone H1, forming a “beads-on-a-string” structure under electron microscopy observation (Luger et al., 2012; Rutowicz et al., 2019). Further compaction results in distinct higher-order structures. Less condensed or open chromatin regions referred to as euchromatin are rich in transcriptionally active genes, and more condensed or closed chromatin regions referred to as heterochromatin are majorly transcriptional inert and often associated with repeat sequences and transposable elements (Vergara and Gutierrez, 2017).

Histone tails protruding from the nucleosome core are subjects to different types of post-translational modifications (PTMs), such as methylation (me), acetylation (ac), ubiquitination (ub) and others (Rando, 2012). PTMs may alter histone-histone or histone-DNA interactions that subsequently affect transcription processes, or alternatively PTMs may serve in recruiting other factors participating in transcription (Krajewski, 2022). In plants, it is generally known that acetylation of histones H3 and H4 (H3ac and H4ac), trimethylation on lysine 4 and lysine 36 of H3 (H3K4me3 and H3K36me3) and monoubiquitination of H2B (H2Bub1) are associated with transcriptional activation, whereas H3K9me2, H3K27me3 and H2Aub1 are associated with silencing/repression of transcription (Alvarez et al., 2010). Transcriptional reprogramming within the chromatin context constitutes a central part of plant defense mechanisms (Tsuda and Somssich, 2015; Birkenbihl et al., 2017).

Treatment of plants with SA or the synthetic SA-analogue benzothiadiazole (BTH), which triggers an intensified response of defense genes to secondary stress, can induce H3/H4ac and H3K4me2/3 levels at chromatin regions within promoters of genes encoding multiple SA pathway-related WRKY transcription factors. These modifications were blocked in priming-deficient plants carrying *npr1* mutations. BTH did not directly activate the expression of these defense genes, which still remained inactive, but these changes contributed to a faster and stronger expression after subsequent infection with *Pseudomonas syringae* pv maculicola (Jaskiewicz et al., 2011). In the same line of histone modifications involved in priming, SAR in Arabidopsis has been associated with activation-related histone PTMs and the priming of SA-dependent defense genes (Conrath et al., 2015). As a synthetic priming activator, non-protein amino acid β-aminobutyric acid (BABA) protects Arabidopsis from oomycete pathogen *Peronospora parasitica* infection by activating plant innate defense mechanisms, such as callose deposition and HR. BABA can still offer a complete protective effect against *Peronospora parasitica* even in plants lacking of SA, JA and ET signaling pathways. Similar to BTH, BABA treatment does not directly induce mRNA accumulation of the relevant defense genes, but can enhance the induction of corresponding genes after stimulation, resulting in stronger resistance (Zimmerli et al., 2000). BABA can also enhance the plant resistance to hemibiotrophic bacteria *Pseudomonas syringae* pv*. tomato* (*Pst*) DC3000 and necrotrophic bacteria *Pectobacterium carotovorum* ssp. carotovorum (*Pcc*) *via* priming mechanism (Ton et al., 2005; Po-Wen et al., 2013). After BABA treatment, the increased responsiveness of PTI-responsive genes to necrotrophic bacteria or relevant MAMP is mediated by histone H3K9ac/K14ac and histone H3K4me2. Correspondingly, PTI-mediated callose deposition and immunity to *Pcc* are also enhanced in BABA-treated Arabidopsis (Po-Wen et al., 2013). The treatment of BABA and 2,6-dichloroisonicotinic acid (INA, the functional synthetic analog of SA) in common bean (*Phaseolus vulagris*) induced plant resistance to *Pseudomonas syringae* pv. *phaseolicola* infection, and changes in H3K4me3 and H3K36me3 markers occur in the promoter-exon regions of defense-related genes (Martinez-Aguilar et al., 2016). In addition, Arabidopsis plants exposed to repetitive heat, cold, or salt stress are more resistant to virulent bacteria than Arabidopsis grown in a more stable environment. Plant resistance is increased by the priming of Arabidopsis PTI-responsive genes and PTI-mediated callose deposition (Singh et al., 2014a).

**Figure 1:**
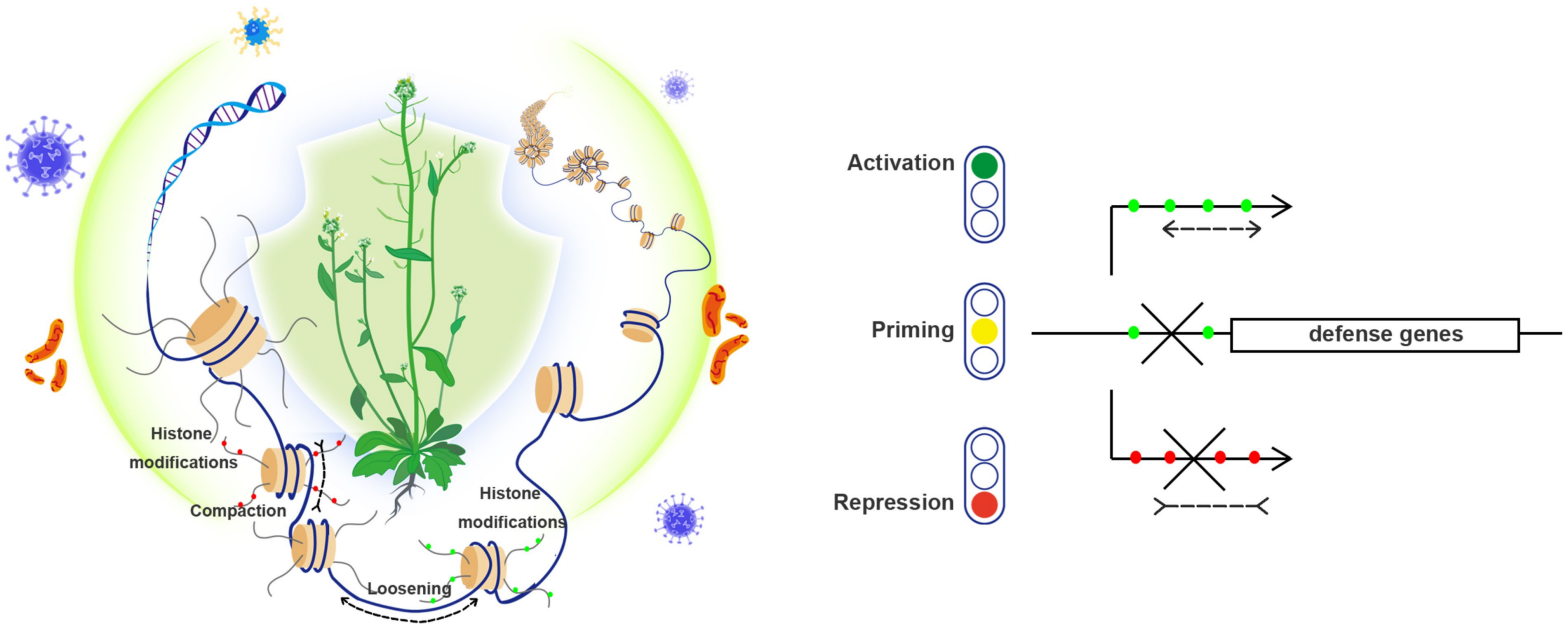
Both histone modification and chromatin remodeling are implicated in plant resistance to pathogens. (Left) Plant nucleosomes are widely subjected to various histone modifications, including repressive histone methylation (such as H3K27me3, labeled as red dots) and permissive histone methylation and acetylation (such as H3K4me3 and H3K14ac, labeled as green dots). Chromatin remodeling includes nucleosome sliding (compaction or loosening of neighboring nucleosomes) and histone variant exchanges. These epigenetic changes coordinate to shape local chromatin, which act as important mechanisms underlying transcriptional reprogramming, and effectively contribute to plant resistance (illustrated by an Arabidopsis plant under protection) to diverse pathogens (virus, bacteria and fungi). (Right) Different transcriptional states of defense genes are illustrated by: gene activation (green light) that is often associated with permissive histone modifications (green dots) and loosened chromatin structure, priming state (yellow light) where permissive histone modifications can also be found but transcription is poised yet uninitiated, and gene repression (red light) that is generally accompanied with repressive histone modifications (red dots) and compacted chromatin structure.

Histone modifications are dynamically regulated by “writers” that are responsible for the establishment of modifications (such as acetyltransferases, methyltransferases, ubiquitin-ligases, etc.) and by “erasers” that are responsible for the removal of modifications (such as deacetylases, demethylases, deubiquitinases, etc.). In addition, histone modifications are specifically recognized by “readers,” which can help to assemble appropriate transcriptional machineries and/or chromatin-remodeling factors or complexes (CRCs). These catalytic factors/complexes utilize the energy from ATP hydrolysis to drive nucleosome sliding along DNA, to alter nucleosome occupancy and positioning, and/or to mediate nucleosomes removal and replacement by variant histones. Variant histones differ from canonical histones at primary amino acid sequence levels and display significant diversity in genome-wide chromatin distribution and PTMs, thus playing critical roles in regulating genome functions. Frequently, chromatin-remodeling factors work together with histone chaperones, which act in avoiding nonspecific interactions between histone and DNA molecules. More information about these above-mentioned histone/chromatin modifiers and their functions in plant growth and development can be found in several recent review articles (Han et al., 2015; Mehta and Jeffrey, 2015; Kang et al., 2020; Borg et al., 2021; Hu and Du, 2022; Shang and He, 2022). Here below, we focus our review on their implication specifically in plant response against pathogens (Figure 1).

## Histone modification regulators involved in plant response to pathogens

### Histone lysine methyltransferases and demethylases

Typically, all known plant histone lysine methyltransferases (HKMTs) contain an evolutionarily conserved SET [named after Su(var)3–9, Enhancer-of-zeste, and Trithorax] domain. Their specificities to different lysine residues (e.g., H3K4, H3K9, H3K27 and H3K36) are determined by distinct SET domains as well as surrounding sequences. Plant histone lysine demethylases (HKDMs) comprise two families: lysine-specific demethylase (LSD1) homologues and Jumonji-C (JmjC) domain-containing proteins (JMJs). So far, a number of HKMTs and HKDMs were reported as involved in plant responses to pathogens (Table 1).

**Table 1:** Histone methyltransferases and demethylases implicated in plant response to pathogens.

Site	Modifiers		Function	Transcription-affected genes	Reference
H3K4	Writers	ATX1(me3)	Activation	*WRKY70, PR1* (SA/JA-pathway genes)	Alvarez-Venegas et al., 2007
		ATXR7(me3)	Activation	*SNC1, RPP4* (NLR genes)	Xia et al., 2013
	Erasers	FLD (me1/2)	Repression	*WRKY38/53/65* (negative regulators in plant immunity response)	Singh et al., 2014b
		LDL1/LDL2(me1/2)	Repression	*WRKY22/40/70, PR1*	Noh et al., 2021
		AtJMJ14(me3)	Repression	*SNI1*	Li et al., 2020
		OsJMJ704(me2/3)	Repression	*NRR, OsWRKY62, Os-11 N3* (rice defense negative regulator genes)	Hou et al., 2015
H3K9	Writers	AtKYP(me2)	Repression	Diverse genes implicated in pathogen-induced PCD.	Dvořák Tomaštíková et al., 2021
				TGS pathway to silence invading DNA viruses, as well as directly control viral chromatin	Castillo-González et al., 2015
		AtSUVH5/6(me2)	Repression	many *PRR/NLR* genes	Cambiagno et al., 2021
				Erasers	AtIBM1(me2)	Activation	*FRK1* (PTI marker gene), *PR1, PR2*	Chan and Zimmerli, 2019
	AtJMJ27(me1/2)	Activation	*PR1, PR3, PR4, PR5*		Dutta et al., 2017
	H3K27	Writers	AtCLF(me2/3)	Repression	Diverse genes implicated in pathogen-induced PCD.	Dvořák Tomaštíková et al., 2021
AtSWN(me2/3)			Repression			
Erasers		AtREF6(me2/3)	Activation	many *PRR/NLR* genes	Cambiagno et al., 2021
		OsJMJ705(me2/3)	Activation	diverse biotic stress-responsive genes	Li et al., 2013
H3K36	Writers	AtSDG8(me2/3)	Activation	*LAZ5* (RPS4-like *R* genes)	Palma et al., 2010
				*ERF1, MYC2, PDF1.2a, VSP2* (JA/ET-responsive genes)*, MKK3, MKK5* (mitogen-activated protein kinase genes)	Berr et al., 2010
				*PR1, PR2, NPR1* (SA-pathway genes)	Zhang et al., 2020b
				*CCR2, CER3* (defense genes)	Lee et al., 2016
		SlSDG33/34(me3)	Activation	*SlERF3, SlRCD1, SlTOPLESS, SlPUB23* (negative regulatory factors)	Bvindi et al., 2022

The transcription of *WRKY70*, a transcriptional factor gene acting at the crosstalk of both SA and JA signaling pathways in Arabidopsis, relies on H3K4me3 deposited by ARABIDOPSIS HOMOLOG OF TRITHORAX 1 (ATX1, Alvarez-Venegas et al., 2007). ATX1 also participates in the transcriptional initiation of the defense response gene *PR1*, keeping it under an ‘actively’ modified chromatin state to respond rapidly in transcription when needed. Correspondingly, *atx1* mutant plants display reduced *PR1* gene expression and reduced resistance to the hemibiotrophic bacteria *Pst* DC3000 (Alvarez-Venegas et al., 2007). Although the mechanism of ATX1 targeting *WRKY70* and *PR1* remains largely unclear, there have been good advances in the targeting mechanism of its homologue ATX-RELATED 7 (ATXR7). ATXR7 binds to the plant-specific protein MODIFIER OF SNC1 9 (MOS9) to modulate *via* H3K4 methylation the transcription of *SUPPRESSOR OF NPR1-1, CONSTITUTIVE 1* (*SNC1*) and *RECOGNITION OF PERONOSPORA PARASITICA 4* (*RPP4*), which encode plant NLR proteins acting as intracellular sensors to detect pathogen effectors and trigger immune response, playing thus an important role in Arabidopsis resistance to oomycete pathogen *Hyaloperonospora arabidopsidis* Emwa1 (Xia et al., 2013). Arabidopsis *FLOWERING LOCUS D* (*FLD*) encoding an LSD1-family HKDM involved in removing H3K4me1/2 was reported as necessary for response to SAR signals, guiding systematic accumulation of SA and enhancement of disease resistance (Singh et al., 2013, 2014b). *LSD1-LIKE 1* (*LDL1*) and *LDL2* involved in removing H3K4me1/2 were also reported to play roles in plant immunity to infections by several *Pst* strains. Compared to wild-type plants, H3K4me1 strongly accumulates at the promoter regions of many defense-related genes in the *ldl1 ldl2* double mutant, indicating that these two HKDMs are involved in controlling the transcription of a group of defense-related genes (Noh et al., 2021). In addition, the HKDM JMJ14 plays a role in local and systemic Arabidopsis plant immune responses by removing pathogen-induced H3K4me3 enrichment and regulating defense genes involved in hormone (such as SA)-mediated defense pathways (Li et al., 2020). In rice, transcription of 15 *JmjC* genes was found induced after plant infection by the bacterial blight disease pathogen *Xanthomonas oryzae* pv. oryzae (*Xoo*). Among them, *JMJ704* inhibits the transcription of negative defense regulator genes, such as *NRR*, *OsWRKY62* and *Os-11 N3*, by reducing H3K4me2/3 levels, and thus positively regulates rice defense responses (Hou et al., 2015). Nonetheless, the specificity of these different HKDMs activities and the potential redundancy of their roles in plant immunity still remain largely obscure and await future investigations.

H3K36 methylation is also involved in the transcriptional regulation of *R* genes. In Arabidopsis, SET-DOMAIN GROUP 8 (SDG8) is the major HKMT catalyzing H3K36me2/3 (Xu et al., 2008), and SDG8 is necessary for H3K36me3 deposition at *LAZARUS 5* (*LAZ5*) that encodes a RPS4-like R protein (Palma et al., 2010). In addition, SDG8 plays a vital role in plant defense against fungal pathogens by modulating a range of genes in JA and/or ET signaling pathways. Loss-of-function of *SDG8* results in mutant plants exhibiting decreased resistance to the fungal pathogens *Alternaria brassicicola* and *Botrytis cinerea* (Berr et al., 2010) as well as to the bacteria pathogen *Pst* DC3000 (Zhang et al., 2020b). Consistently, several defense genes in wild-type plants infected with fungal pathogens or methyl JA treatment showed dynamic changes in SDG8-dependent histone H3K36 methylation. It was proposed that SDG8-mediated histone H3K36 methylation may act as a memory mechanism for a range of defense genes including those involved in JA/ethylene signaling pathway, helping them to achieve rapid transcription upon fungal infection or JA treatment (Berr et al., 2010). Meanwhile, SDG8 can also physically interact with the C-terminal domain of RNA polymerase II (RNAPII), linking H3K36me3 deposition with RNAPII loading, for defense gene transcriptional induction by SA (Zhang et al., 2020b). Lastly, SDG8 was also reported to regulate *CAROTENOID ISOMERASE 2* (*CCR2*) and *ECERIFERUM 3* (*CER3*), which encode proteins to catalyze the biosynthesis of carotenoids and be involved in the biosynthesis of cuticular wax, respectively, and loss of SDG8 resulted in the reduction of lipid accumulation and loss of cuticle integrity leading to impeded plant immunity (Lee et al., 2016). In tomato (*Solanum lycopersicum*, *Sl*), the SDG8-orthologues SlSDG33 and SlSDG34 were required for pathogen- and stress-induced enrichment of H3K36me3 and H3K4me3 at target genes, but their loss increased plant resistance to *Botrytis cinerea* (Bvindi et al., 2022). It was proposed that mutations in Sl*SDG33* and Sl*SDG34* may disrupt permissive transcriptional context promoting expression of negative regulatory factors to allow improvement of plant tolerance without growth trade-offs.

In contrast to the above activation histone methylations, H3K27me2/3 marks the silenced or suppressed state of gene transcription. Arabidopsis LIKE HETEROCHROMATIN PROTEIN 1 (LHP1), which binds to H3K27me3 through its chromodomain and is required for the spreading of H3K27me3, was found to be implicated in the repression of the *MYC2* branch of JA/ET pathway of plant immunity, and its loss-of-function leads to reduced SA content and resistance to *Pst* DC3000 (Ramirez-Prado et al., 2019). Arabidopsis rapidly reprograms transcriptome after programmed cell death (PCD) triggered in response to immunity- and disease-promoting pathogen effectors, and changes in gene expression are associated with H3K27me3. Polycomb Repressive Complex 2 (PRC2) is responsible for H3K27me2/3 deposition and it is SET-domain-containing components CURLY LEAF (CLF) and SWINGER (SWN) have partial non-redundant functions in HR progression after *Pst* DC3000 infection (Dvořák Tomaštíková et al., 2021). The HKDM RELATIVE OF EARLY FLOWERING 6 (REF6)/JMJ12 involved in removing H3K27me2/3 was shown to contribute to the establishment of specific chromatin states in promoting priming of many genes including *PRR* and *NLR* genes that encore immune receptors in Arabidopsis (Cambiagno et al., 2021). In rice, JMJ705 specifically removes H3K27me2/3 and its overexpression results in the preferential activation of biotic stress response genes marked by H3K27me3, which enhances rice resistance to bacterial blight disease pathogen *Xoo* (Li et al., 2013). Notably, JMJ704 and JMJ705 show a different target specificity (H3K4me2/3 vs. H3K27me2/3), although they are jointly involved in the positive regulation of rice defense mechanisms against *Xoo* (Hou et al., 2015).

In addition to H3K27me3, H3K9me2 deposited by the HKMT SU(VAR)3–9 HOMOLOG 4 (SUVH4)/ KRYPTONITE (KYP), along with its homologs SUVH5/6, is also involved in repressing the basal expression of *PRR*/*NLR* genes (Cambiagno et al., 2021). While *PRR*/*NLR* genes determine the plant capability to resist pathogen infections, their excessive activation is detrimental to normal plant growth and development, pointing to a strict modulation role of H3K9 methylation in fine-tuning gene expression in plant immunity. In line with this, the HKDM INCREASE IN BONSAI METHYLATION 1 (IBM1)/JMJ25 and its homologue JMJ27 had been shown as required in removing H3K9me2 to ensure a permissive chromatin context for the correct induction of defense genes (including *PR1*/*2*) for Arabidopsis resistance to *Pst* DC3000 (Dutta et al., 2017; Chan and Zimmerli, 2019).

It is worth noting that epigenetic modifications can also occur in pathogens and contribute to plant-pathogen interactions. It has been reported that in innate immunity against geminivirus SUVH4/KYP binds viral chromatin and controls its methylation to combat virus infection (Castillo-González et al., 2015). The geminivirus-encoded TrAP protein interacts with the catalytic domain of SUVH4/KYP and inhibits its HKTM activity, thus counteracting plant defense, forming an epigenetic regulatory node between the host antiviral defense and virus counter defense (Castillo-González et al., 2015). Soybean (*Glycine max*) root rot pathogen *Phytophthora sojae* evades the soybean *R*-gene *Rps1b* by its PRC2 PsSu(z)12-mediated transcriptional polymorphism in the effector gene *Avr1b* without any sequence variation. The *PsSu(z)12* mutation leads to the loss of H3K27me3 in the *Avr1b* region, increased *Avr1b* expression, and thus the loss of ability to evade the immune recognition by Rps1b and the loss of virulence in soybeans (Wang et al., 2020a). Fungal pathogens secrete effectors that are highly expressed *in planta* but inhibited *ex planta*. It was found that H3K27me3 provides significant local transcriptional repression *ex planta* and is replaced by H3K27ac when highly expressed *in planta*, enabling a strict regulation of effector genes that contribute to host infection (Zhang et al., 2021).

### Histone acetyltransferases and deacetylases

Histone acetylation is catalyzed by histone acetyltransferases (HATs), and removed by histone deacetylases (HDACs; Hollender and Liu, 2008; Chen et al., 2020; Kumar et al., 2021). Based on sequence similarity and domain organization, HATs can be divided into four classes: GENERAL CONTROL NONDEREPRESSIBLE 5 (GCN5)-RELATED ACETYLTRANSFERASE (GNAT, also named HAG), MOZ-YBF2/SAS3-SAS2/TIP60 (MYST, also named HAM), cAMP-RESPONSIVE ELEMENT BINDING PROTEIN (CREB)-BINDING PROTEIN (CBP, also named HAC), and TATA-BINDING PROTEIN ASSOCIATED FACTOR 1 (TAFII250, also named HAF). Plant HDACs can be divided into three classes: RPD3-like, sirtuin-like, and plant-specific Histone Deacetylase 2 (HD2). While RPD3-like HDACs require zinc as a cofactor for deacetylase activity, Sirtuin-like HDACs remove acetyl groups in an NAD^+^-dependent manner. Both HAT-mediated histone acetylation in transcriptional activation and HDAC-mediated histone deacetylation in transcriptional repression are involved in plant responses to pathogens (Table 2).

**Table 2:** Histone acetyltransferases and deacetylases implicated in plant response to pathogens.

Modifiers			Function	Transcription-affected genes	Reference
Writers	HAG	AtGCN5	Activation	*WRKY33, DND2, MYC2* (SA pathway genes)	Kim et al., 2020
				diverse defense genes including *WRKY33, NAC*	Kong et al., 2017
	HAC	AtHAC1/5	Activation	*PR1, PR2*	Jin et al., 2018
				*WRKY53, FRK1, NHL10* (PTI-responsive genes)	Singh et al., 2014a
Erasers	RPD3	AtHDA6	Repression	Diverse defense genes including *PR1, PR2*	Wang et al., 2017
				*CBP60g, SARD1* (SA synthesis)	Wu et al., 2021b
		AtHDA9	Repression	Diverse NLR genes	Yang et al., 2020
		AtHDA19	Repression	SA-pathway genes including *PR1, PR2*	Zhou et al., 2005
					Choi et al., 2012
		OsHDA701	Repression	diverse defense genes in rice	Chen et al., 2022
	Sirtuin	AtSRT2	Repression	*PAD4, EDS5, SID2* (SA synthesis), *PR1*	Wang et al., 2010
	HD2	HD2B	Repression	flg22-regulated genes and diverse biotic stress response genes	Latrasse et al., 2017
		TaHDT701	Repression	*TaPR1, TaPR2, TaPR5* and *TaWRKY45*	Zhi et al., 2020

Exposure of plants to sub-lethal levels of salt, cold or heat can enhance their resistance to infection by pathogens. In such cross-priming, PTI-responsive genes are enriched by histone acetylation associated with their transcriptional activation. Compared to wild-type Arabidopsis plants, mutants of *HISTONE ACETYLTRANSFERASE 1* (*HAC1*), which encodes a HAT belonging to the HAC/CBP class, are impaired in priming PTI responses and fail to exhibit enhanced resistance to bacteria (Singh et al., 2014a). In the SA signaling pathway, HAC1/5 forms together with NPR1, a coactivator complex that is recruited through the transcription factor TGA, to activate *PRs* transcription *via* histone acetylation-mediated epigenetic reprogramming (Jin et al., 2018). HAG1/GCN5 has been considered as a master modulator of plant-environment interaction by inhibiting SA accumulation and SA-mediated immunity (Kim et al., 2020). Being a component of the SPT-ADA-GCN5 ACETYLTRANSFERASE (SAGA) transcriptional co-activator complex, Arabidopsis HAG1/GCN5 acetylates multiple sites of histone H3 (Wu et al., 2021a). While *hag1/gcn5* mutation led to a decrease of H3K14ac at the 5′ end of HAG1/GCN5 down-regulated targets, it also led to an increase of H3K14ac at the 3′ end of HAG1/GCN5 up-regulated targets, revealing a dual role of HAG1/GCN5 in establishing acetylation status of its target genes. Moreover, HAG1/GCN5 also affected H3K9ac and H3K27ac levels (Wu et al., 2021a).

Pathogen effectors may inhibit the activation of plant defense genes by interfering with the function of HAT complex during infection. Indeed, the *Phytophthora sojae* effector PsAvh23 binds the ADA2 subunit of SAGA complex and interrupts the interaction between ADA2 and GCN5, thus leading to the dysregulation of defense-related genes and increased soybean plant susceptibility to the pathogen infection (Kong et al., 2017). *PsGCN5*-silenced mutant strains show impaired virulence likely due to their higher sensitivity to hydrogen peroxide (H_2_O_2_), a major oxidative product induced in host plant during early immune response, and to their incapability to inhibit ROS production in soybean plants (Zhao et al., 2015). Catalase plays a vital role in protecting cells from oxidative damage by catalyzing H_2_O_2_ degradation into water and oxygen. In the filamentous fungus *Neurospora crassa*, NcGCN5 acts as a positive regulator of *CATALASE-3* transcription *via* H3 acetylation and its deprivation leads to the failure of fungus to effectively resist H_2_O_2_ accumulation (Qi et al., 2018). It becomes evident that GCN5 functions as a major HAT in plants and in pathogens, both can be detrimental for their interactions.

Besides HATs, HDACs also play essential roles in plant-pathogen interactions. Both Arabidopsis HISTONE DEACETYLASE 6 (HDA6) and HDA19, which belong to the RPD3-like class of HDACs, play key regulatory roles in plant responses to pathogens. *HDA19* expression is induced by wound as well as by the *Alteraria brassicicola* and *Pst* pathogens (Zhou et al., 2005). Loss of HDA19 increases SA accumulation and the expression of related genes as well as *PR* genes, resulting in enhanced resistance to *Pst* (Kim et al., 2008). HDA19 binds directly to *PR1*/*2* promoter and then deacetylates histones, ensuring low basal expression levels of *PR1*/*2* genes under unchallenged conditions as well as avoiding an overstimulated expression of these genes during the defense response to pathogen attack (Choi et al., 2012). Similarly, HDA6 binds and represses the expression of pathogen-responsive genes (including *PR1*/*2*, etc.) under both normal growth conditions and pathogen infections (Wang et al., 2017). HDA6 inhibits SA biosynthesis by directly controlling the expression of *CBP60g* and *SARD1* (Wu et al., 2021b). Remarkably, the opposing activities of HDA6 and GCN5 also maintain acetylation homeostasis of TOPLESS (TPL), a non-histone protein, which represses the master transcriptional factor MYC2 involved in plant immunity (An et al., 2022).

The geminivirus-encoded protein V2 protein interacts with *Nicotiana benthamiana* NbHDA6, and competes the NbMET1-NbHDA6 interaction, thus interfering the recruitment of NbMET1 by NbHDA6 to downregulate the TGS-triggered viral DNA genome methylation, which eventually increases host susceptibility to geminivirus infection (Wang et al., 2018). UvSec117, a key effector secreted by *Ustilaginoidea virens*, targets the rice RPD3-like class HDAC OsHDA701 to the nucleus to reduce H3K9ac and interfer with the activation of H3K9ac-marked defense gene transcription, which eventually promotes pathogen infection (Chen et al., 2022). Overexpression of BcRPD3 in *Botrytis cinerea* led to a significant decrease in the levels of histones H3 and H4 acetylation at virulence genes, resulting in a slight delay in fungus vegetative growth and infectious structures formation and significantly weakened virulence (Zhang et al., 2020a).

In addition to the RPD3-like HDACs, the Sirtuin-like HDAC AtSRT2 also has a negative regulatory role in Arabidopsis basal resistance against *Pst* DC3000, associated with the repression of SA pathway-related genes such as *PHYTOALEXIN DEFICIENT 4* (*PAD4*) and *PR1* (Wang et al., 2010). The HD2-class HDAC AtHD2B was identified as targeted by the MAPK-type kinase MPK3 to re-localize from nucleolus to nucleoplasm to repress the transcription of flg22-regulated genes and biotic stress-responsive genes (Latrasse et al., 2017). More recently, another HD2-class HDAC AtHD2C was identified as a positive regulator of plant resistance to *Cauliflower mosaic virus* (CaMV) infection (Li et al., 2021). AtHD2C is involved in silencing viral genome by decreasing histone acetylation on viral minichromosome. As counteraction, the CaMV P6 protein physically interacts with AtHD2C and interferes with the interaction between AtHD2C and HDA6, leading to HDAC dysfunction and thus promoting CaMV infection. Correspondingly, the susceptibility to CaMV infection is increased in the *hd2c* mutants (Li et al., 2021). The wheat (*Triticum aestivum*) HD2-class HDAC TaHDT701 can stabilize the interaction between TaHDA6 and HIGH EXPRESSION OF OSMOTICALLY RESPONSIVE GENES 15 (TaHOS15), forming a larger HDAC complex involved in plant resistance to wheat powdery mildew (Zhi et al., 2020). Notably, Arabidopsis HOS15 homolog physically interacts with HDA9, another RPD3-like HDAC in Arabidopsis, and function together as the negative regulators of one-third of diverse NLR genes in Arabidopsis likely through altering the local H3K9 acetylation (Yang et al., 2020). Whether or not an Arabidopsis HD2 member also participates together with HOS15-HDA9 to form a larger HDAC complex in plant immunity still remains unclear yet.

Lastly, it is worthy to note that enzymatic inhibitors of HDACs also play essential functions in plant-pathogen interactions. The cyclic tetrapeptide HC-toxin (HCT) from the filamentous fungus *Cochliobolus* (formerly *Helminthosporium*) *carbonum* inhibits enzymatic activities of numerous HDACs *in vitro* (Brosch et al., 1995). HCT treatments of maize (*Zea mays* L.) led to hyperacetylation of histones H3 and H4 as well as non-histone proteins including the TPL-homolog RAMOSA1 ENHANCER LOCUS 2 (REL2; Ransom and Walton, 1997; Walley et al., 2018). It is likely that HCT promotes the establishment of pathogenic compatibility between *Cochliobolus carbonum* and maize by interfering with the reversible acetylation of histones and/or non-histone transcription regulators. It is known that pathogen-induced SA signaling promotes biosynthesis of nitric oxide (NO), and that NO can physically inhibit enzymatic activities of various HDACs. A more recent study in Arabidopsis demonstrated that treatment with S-nitrosoglutathione (GSNO), the physiological NO donor, increased global levels of all examined acetylation markers of histones H3 and H4 (Mengel et al., 2017). Genome-wide H3K9/14 ac-profiles revealed that NO-regulated histone acetylation occurs at many genes involved in plant defense response, suggesting that NO-mediated inhibition of HDAC activities favors hyperacetylation and transcription of these specific genes (Mengel et al., 2017). Sodium valproate (SV) can enhance acetylation of many histone H3 lysines (H3K9ac, H3K14ac and H3K56ac) and can act as a fungicide to make tomato plants more resistant to *Botrytis cinerea* infection (Xu et al., 2022). SV treatment altered the expression levels of several genes associated with *Botrytis cinerea* virulence as well as tomato immune response, indicating that changes of acetylation level within chromatin of both plants and pathogens are involved in their interplay.

### Other histone modifiers

Arabidopsis RING E3 ligase HISTONE MONOUBIQUITINATION1 (HUB1) and HUB2 mediate histone H2B monoubiquitination (H2Bub1). *HUB1*-deficient Arabidopsis mutants exhibited higher sensitivity to *Botrytis cinerea* and *Alternaria brassicicola*, while *HUB1* overexpression showed resistance to *Botrytis cinerea* (Dhawan et al., 2009). HUB1/HUB2 mediate H2Bub1 directly at the *R*-gene *SNC1* to induce its expression, which is associated with autoimmune phenotypes and enhanced disease resistance (Zou et al., 2014). The HUB1/HUB2 homologues in tomato, SlHUB1/SlHUB2, have similar H2B monoubiquitination E3 ligase activity *in vitro*, and their silencing leads to increased plant sensitivity to *Botrytis cinerea* (Zhang et al., 2015). Correspondingly, gene expression in the SA-mediated signaling pathway was significantly upregulated, while gene expression in the JA/ET-mediated signaling pathway was significantly reduced, in *SlHUB1*/*HUB2-*silenced plants. Thus, SlHUB1/SlHUB2 likely play a role in defense response against *Botrytis cinerea* by adjusting the balance between SA and JA/ET-mediated signaling pathways (Zhang et al., 2015).

The lysine 2-hydroxyisobutyrylation (Khib) is a more recently discovered histone modification, and is widely distributed as an active histone marker associated with gene transcriptional activation (Dai et al., 2014; Huang et al., 2018). In rice, histone Khib is involved in regulating the expression of *R* genes. The ascomycete *Ustilaginoidea virens* can regulate Khib in rice where most Khib sites in histone H3 were downregulated during infection (Chen et al., 2021). The rice RPD3-like HDAC HDA705 is involved in removing Khib, negatively regulating plant resistance to a wide range of pathogens including *Ustilaginoidea virens*, while knockingout of *HDA705* enhances disease resistance to these pathogens (Chen et al., 2021).

## Chromatin-remodeling factors involved in plant immunity

Genome-wide transcriptional analysis reveals that SAR is involved in the processes of transcriptional reprogramming (Thibaud-Nissen et al., 2006; Wang et al., 2006), and chromatin remodeling activity is known to be extensively involved in the corresponding processes (van den Burg and Takken, 2009; Ramirez-Prado et al., 2018). All chromatin-remodeling factors show homology to the yeast SNF2 ATPase protein and contain a common Snf2 domain. They can modulate the structural and dynamic properties of chromatin, thereby influence a wide range of nuclear processes (Flaus et al., 2006). Chromatin-remodeling factors can be divided into four subfamilies based on the catalytic Snf2 domain and other accessory domains: Switch/Sucrose on-fermentable (SWI/SNF), Inositol auxotrophy 80 (INO80), Imitation switch (ISWI), and Chromodomain helicase DNA-binding (CHD; Clapier et al., 2017). These ATPases usually perform remodeling functions individually or in the form of multi-subunit CRCs (Clapier et al., 2017; Hannan Parker et al., 2022), and members from all the four subfamilies were found involved in plant immunity (Table 3).

**Table 3:** Chromatin-remodeling factors implicated in plant response to pathogens.

Subfamilies	Remodelers	Activities	Function	Transcription-affected genes	Reference
**SWI/SNF subfamily**					
ATPase	SYD	Nucleosome relocation	Activation	JA/ethylene pathway genes including *PDF1.2a*	Walley et al., 2008
			Repression	*SNC1* (NLR protein), *PR1, PR2*	Johnson et al., 2015
subunit	SWP73A		Repression	*NLRs* or *CDC5*	Huang et al., 2021
**INO80 subfamily**					
SWR1-C-ATPase	PIE1	H2A.Z exchange	Repression	SAR-dependent genes including *NPR1*	March-Díaz et al., 2008
SWR1-C-subunit	SWC6		Activation	pathogen-induced *PR1* and *PR5* activation (ARP6 plays a repressive role instead in this case)	Berriri et al., 2016
SWR1-C-subunit	ARP6		Repression	Genes involved in PTI responses	Cheng et al., 2013
Functionally related histone chaperones	NRP	Counteract SWR1-C	Activation	Diverse *WRKY* genes and *PR* genes	Barna et al., 2018
ATPase	CHR19	Nucleosome relocation	Repression	SA/JA-pathway genes	Kang et al., 2022
**ISWI subfamily**					
ATPase	CHR11	Nucleosome relocation	Repression	Diverse defense response genes including *PR1, PR2*	Liu et al., 2021
ATPase	CHR17				
**CHD subfamily**					
ATPase	CHR5	Nucleosome relocation	Activation	*SNC1* (NLR protein)	Zou et al., 2017

### SWI/SNF subfamily

In *Arabidopsis*, BRAHMA (BRM) and SPLAYED (SYD) are highly conserved SWI/SNF ATPases, which exhibit similar as well as distinct roles in regulating plant developmental processes (Bezhani et al., 2007). SYD can be directly recruited to the promoter region of target genes to regulate the expression of multiple defense genes downstream of JA and ET signaling pathways. SYD is involved in defense against *Botrytis cinerera*, which is associated with the activation of JA-pathway defense gene *PLANT DEFENSIN1.2a* (*PDF1.2a*; Walley et al., 2008). In addition, together with other proteins, SYD co-represses *SNC1* transcription at the chromatin level, and this regulatory role is important for fine-tuning the expression of NLR-encoding genes to prevent adverse autoimmunity (Johnson et al., 2015). In contrast, the *syd* mutant barely shows modified resistance to *Pst*. This mild effects of *syd* may be partly explained by its redundancy with *BRM* in SA-mediated signaling, because both *SYD* and *BRM* have been shown to act on shared as well as unique target genes (Bezhani et al., 2007; Wu et al., 2012). Yet, an overlapping or specific role of *BRM* in plant immunity still remains to be elucidated.

NLR-mediated immunity is precisely controlled to avoid adverse autoimmunity. SWI/SNF-ASSOCIATED PROTEINS 73 (SWP73A) is a subunit of the SWI/SNF CRC (Jerzmanowski, 2007; Hopfner et al., 2012). SWP73A can directly repress the expression of multiple *NLR*s by binding to their promoters, or inhibit the expression of gene encoding the key RNA splicing regulatory protein CELL DIVISION CYCLE 5 (CDC5), thereby affecting the alternative splicing of *NLR*s. Upon the *Pst* infection, bacteria-induced small RNAs silence *SWP73A* to activate the correct induction and alternative splicing of *NLR* genes involved in the ETI pathway. Thus, SWP73A acts as a negative regulator of gene expression, and inhibits the endogenous immune response of plants to avoid autoimmunity in the absence of pathogens (Huang et al., 2021).

### INO80 subfamily

The INO80 subfamily comprises two highly conserved groups: INO80 and SWI2/ SNF2-related 1 (SWR1). Both INO80 and SWR1 ATPases act as scaffold and core catalytic subunit for assembling protein complexes with other accessary proteins, the INO80-Complex (INO80-C) and SWR1-C, respectively. INO80-C and SWR1-C comprise their specific subunits, such as ACTIN-RELATED PROTEIN 5 (ARP5) and ARP6, respectively (Wang et al., 2019). Meanwhile, they also share several common subunits including RUVB-LIKE 1 (RVB1), which belongs to a large family of proteins called AAA+ class of chaperone-like ATPases (Neuwald et al., 1999). RVB1 in plants, also known as RESISTANCE TO PSEUDOMONAS SYRINGAE PV MACULICOLA INTERACTOR 1 (RIN1), negatively regulates the expression of many *R*-genes. RVB1/RIN1 interacts with CC-type NLR protein RESISTANCE TO *PSEUDOMONAS SYRINGAE* PV *MACULICOLA* (RPM1) and TIR-type NLR protein RPP5 to participate in plant defense pathways. Reduction of *RVB1/RIN1* mRNA level results in the measurable increase of two *R*-dependent responses without constitutively activating defense responses (Holt 3rd et al., 2002).

SWR1-C can mediate the replacement of histone H2A by the variant histone H2A.Z and is required for repression of SA-dependent defense genes (March-Díaz et al., 2008). All components of Arabidopsis SWR1-C have been identified (Luo et al., 2020), and mutations in the genes encoding catalytic core subunit PHOTOPERIOD-INDEPENDENT EARLY FLOWERING 1 (PIE1) and subunits SWR1-C SUBUNIT 6 (SWC6) and ARP6 impair H2A.Z replacement, leading to the abnormal expression of a large number of genes that overlaps with the consequence of endogenous *H2A.Z* gene dysfunction in plants. Many of the misregulated genes are involved in SA-dependent immune pathways. Functional knockout of these genes leads to constitutive activation of SA-mediated defense responses, and these mutants exhibit abnormal SA biosynthesis, overexpression of many SA-responsive genes, and spontaneous cell death under normal conditions, thus altering plant resistance to both biotrophic and necrotrophic pathogens (Berriri et al., 2016). Genome-wide expression analyses further revealed that these SWR1-C subunits have complex and specific functions in defense gene regulation, and PIE1 may play an important role in the regulation of crosstalk between different defense signaling pathways (Berriri et al., 2016).

SWR1-C also interacts with histone chaperone NUCLEOSOME ASSEMBLY PROTEIN-RELATED PROTEIN (NRP) in the H2A.Z regulation. NRP is a histone chaperone exhibiting higher affinities for H2A/H2B than H3/H4, while it can also effectively bind H2A.Z *in vivo*. Under normal growth conditions, NRP proteins cause a decrease in H2A.Z-containing nucleosomes in Arabidopsis chromatin and thereby counteract SWR1-C activity in the regulation of gene expression (Wang et al., 2020b). Pathogen infection tests have revealed that aging of plant tissues favors necrotrophs, while juvenile plants generally favor biotrophic pathogens (Glazebrook, 2005; Barna et al., 2012). Tissue damage caused by *Pst* was found in *nrp* mutant plants at early aging (Barna et al., 2018). The *nrp* mutants are more susceptible to infection by *Sclerotinia sclerotiorum* and *Pst*, while *NRP* overexpression increases resistance to these pathogens (Barna et al., 2018).

Plants preferentially activate ETI signaling at relatively low temperatures (10–23°C), while switching to PTI signaling at moderately elevated temperatures (23–32°C; Cheng et al., 2013). Elevated temperature suppresses plant immunity (Wang et al., 2009; Alcazar and Parker, 2011). SWR1-C-mediated H2A.Z-containing nucleosome dynamics have been shown to underlie temperature responses in *Arabidopsis*, which enrich in the genes that respond to environmental and developmental stimuli, such as those involved in immune and temperature responses, and play important roles in controlling gene expression (Kumar and Wigge, 2010; Coleman-Derr and Zilberman, 2012). The *arp6* and *h2a.z* mutants phenocopy plants grown at elevated temperatures, and exhibit enhanced PTI and yet reduced ETI responses (Cheng et al., 2013). Thus, SWR1-C and H2A.Z may participate in the plant resistance to different pathogens in a manner influenced by temperature fluctuation.

In Arabidopsis, another ATPase of INO80 subfamily, CHROMATIN REMODELING 19 (CHR19), has a conserved ATP-dependent nucleosome sliding activity. Loss-of-function of *CHR19* causes substantial changes in genome-wide nucleosome positioning and occupancy (Kang et al., 2022). CHR19 is involved in the repression of SA/JA stress-responsive genes under normal growth conditions, coordinating plant growth balance between development and stress response, and contributing to the improvement of plant resistance to fungal pathogens. Moreover, *chr19* mutant displayed higher susceptibility to the JA pathway-defended necrotrophic fungal pathogen *Botrytis cinerea* (Kang et al., 2022).

### ISWI and CHD subfamilies

Arabidopsis ISWI proteins are encoded by two functionally redundant genes, *CHR11* and *CHR17* (Knizewski et al., 2008; Li et al., 2012; Han et al., 2015). CHR11 and CHR17 are required for evenly spaced nucleosome pattern in gene bodies, and the primary role of ISWI is to slide nucleosomes, but not to affect nucleosome structures and density (Racki and Narlikar, 2008; Li et al., 2014). They are negative regulators of plant disease resistance. In the absence of pathogen infection, loss-of-function of *CHR11* and *CHR17* leads to the upregulation of a large set of defense response genes. The *chr11* single mutant shows enhanced resistance against *Pst* DC3000 (Liu et al., 2021). The mutation of *PAD4*, which impairs SA accumulation (Jirage et al., 1999), specifically rescued the upregulation of both defense gene expression and resistance to *Pst* DC3000 in the *chr11 chr17* double mutant (Liu et al., 2021).

Arabidopsis CHD1 is encoded by *CHR5*, which promotes the expression of seed maturation genes and participates in the regulation of plant growth and development (Zou et al., 2017). *CHR5* is required for the upregulation of the intracellular immune receptor gene *SNC1* likely *via* the regulation of nucleosome occupancy in the promoter region. Intriguingly, however, *CHR5* acts as a positive regulator of the *SNC1*-independent plant immune pathway against *Pst*, implying its role in regulation of other defense genes (Zou et al., 2017).

## Conclusion and perspectives

Studies in the past 20 years have greatly advanced our understanding of how diverse histone modifications and chromatin remodeling affect plant defenses against pathogens. Accumulating evidences have revealed that both plants and pathogens rely on epigenetic machineries for their proper development and metabolism. These machineries and associated mechanisms are collectively used to establish or maintain specific transcriptional states, thereby effectively activating or repressing gene transcription, or establishing priming states and/or immune memory. Meanwhile, in addition to the characterization of conserved chromatin modifiers, the study of various environmental factors as well as synthetic chemicals and drugs and pathogenic metabolites that directly or indirectly participate in the establishment, maintenance or change of epigenetic states greatly helps to understand the molecular mechanisms underlying plant responses against pathogens.

The regulation of plant immunity at the chromatin level is located most likely at the end step of signaling pathways, which ultimately affects the plant transcriptome, proteome and metabolome. Albeit the identification of a large number of chromatin modifiers involved in plant defense against pathogens, most of the current studies are limited in the analysis of individual modifiers, and their focus is often confined within the transcriptional regulation of several star defense genes. Although these analyses have successfully revealed many regulatory mechanisms in well-known pathways and uncovered the convergence or interplay of different mechanisms on some target genes, they often ignore the spatiotemporal and dynamic nature of pathogenic infection as well as the complexity of plant-pathogen interplays.

Future studies may also be needed to better integrate histone modifications and chromatin remodeling together with other epigenetic regulations such as DNA methylation and small RNAs. High-throughput profiling data and single-cell sequencing techniques would provide a better spatiotemporal resolution for studying plant-microbial interactions to unravel genome-wide epigenetic changes and complex network regulation underlying this important dynamic process. Such knowledge will be helpful for better understanding the initiation of defense gene transcription and plant immune activation as well as the molecular mechanisms that balance plant stress resistance with normal plant growth and development. The molecular and system-level knowledge will be essential for designing crops that enhance immune responses for improving plant defenses while avoiding or minimizing undesirable side effects, such as reduced plant growth and yield.

## Author contributions

YZ and W-HS conceived the idea. HK, TF, JW and YZ wrote the first version. W-HS revised and finalized the manuscript. All authors contributed to the article and approved the submitted version.

## Funding

This work was financially supported by the National Natural Science Foundation of China (Grant NSFC 32070201 and 31671341) to YZ, and China Postdoctoral Science Foundation (No. 2022 M710782) to HK. W-HS received supports from the Centre National de la Recherche Scientifique (Laboratoire Interna-tional Associé Plant Epigenetic Research) and the Agence National de la Recherche (ANR-19-CE20-0018).

## Conflict of interest

The authors declare that the research was conducted in the absence of any commercial or financial relationships that could be construed as a potential conflict of interest.

## Publisher’s note

All claims expressed in this article are solely those of the authors and do not necessarily represent those of their affiliated organizations, or those of the publisher, the editors and the reviewers. Any product that may be evaluated in this article, or claim that may be made by its manufacturer, is not guaranteed or endorsed by the publisher.

## Abbreviation

CRC: chromatin-remodeling complex
DAMP: damage-associated molecular pattern
ETI: effector-triggered immunity
ETS: effector-triggered susceptibility
HR: hypersensitive response
IR: induced resistance
JA: jasmonic acid
MAMP: microbial-associated molecular patterns
NB-LRR: nucleotide-binding leucine-rich repeat
NLR: NB-LRR-containing R protein
PAMP: pathogen-associated molecular patterns
PRRs: pattern recognition receptors
PTI: pattern-triggered immunity
PTMs: post-translational modifications
ROS: reactive oxygen species
SA: salicylic acid
SAR: systemic acquired resistance.
